# Telomere-related genes as potential biomarkers to predict endometriosis and immune response: Development of a machine learning-based risk model

**DOI:** 10.3389/fmed.2023.1132676

**Published:** 2023-03-09

**Authors:** He Zhang, Weimin Kong, Yunkai Xie, Xiaoling Zhao, Dan Luo, Shuning Chen, Zhendong Pan

**Affiliations:** Department of Gynecological Oncology, Beijing Obstetrics and Gynecology Hospital, Beijing Maternal and Child Health Care Hospital, Capital Medical University, Beijing, China

**Keywords:** telomere, endometriosis, immune response, machine learning, risk model

## Abstract

**Introduction:**

Endometriosis (EM) is an aggressive, pleomorphic, and common gynecological disease. Its clinical presentation includes abnormal menstruation, dysmenorrhea, and infertility, which seriously affect the patient's quality of life. However, the pathogenesis underlying EM and associated regulatory genes are unknown.

**Methods:**

Telomere-related genes (TRGs) were uploaded from TelNet. RNA-sequencing (RNA-seq) data of EM patients were obtained from three datasets (GSE5108, GSE23339, and GSE25628) in the GEO database, and a random forest approach was used to identify telomere signature genes and build nomogram prediction models. Gene Ontology, Kyoto Encyclopedia of Genes and Genomes, and Gene Set Enrichment Analysis were used to identify the pathways involved in the action of the signature genes. Finally, the CAMP database was used to screen drugs for potential use in EM treatment.

**Results:**

Fifteen total genes were screened as EM–telomere differentially expressed genes. Further screening by machine learning obtained six genes as characteristic predictive of EM. Immuno-infiltration analysis of the telomeric genes showed that expressions including macrophages and natural killer cells were significantly higher in cluster A. Further enrichment analysis showed that the differential genes were mainly enriched in biological pathways like cell cycle and extracellular matrix. Finally, the Connective Map database was used to screen 11 potential drugs for EM treatment.

**Discussion:**

TRGs play a crucial role in EM development, and are associated with immune infiltration and act on multiple pathways, including the cell cycle. Telomere signature genes can be valuable predictive markers for EM.

## 1. Introduction

A common gynecological disease, endometriosis (EM), characterized by chronic pelvic pain, dysmenorrhea, and infertility, affects 10% of women of reproductive age worldwide and dramatically reduces their quality of life (1). EM is defined as the presence of endometrial tissue (glands and mesenchyme) with a growth function outside the uterus. About 1% of EM cases may develop malignant changes (2). Currently, the two main EM treatment types are surgical excision and hormonal drug therapy. These are challenging due to their high recurrence rate and ovarian function suppressive effects, respectively (3). Therefore, newly identifying the molecular features of EM and elucidating its underlying mechanisms will aid development of novel and practical therapeutic approaches.

Telomeres, nuclear protein complexes at the ends of human chromosomes, consist of guanine-rich 5′-TAGGG-3′ repeat sequences and shelter proteins (4). Telomeres are essential for chromosome stability, protecting the genome from nucleolytic degradation, undesired recombination, repair, and end-to-end chromosome fusion (5). Abnormalities in telomeres can lead to many health issues, including tumors, heart disease, and mental health problems (6). Telomere lengths shorten following cell division and some disease states, but are protected by the specialized enzyme telomerase. Human telomerase consists of three core subunits: (1) the telomerase RNA component, (2) the catalytic subunit telomerase reverse transcriptase (hTERT), and (3) the dyskerin protein (7).

Telomerase activity (TA) is a dynamic process in the human endometrium. This process is influenced by the ovarian cycle and hormone levels (8). In particular, the highest TA levels are found in endometrial epithelial cells during the proliferative phase. Elevated TA in endometrial epithelial cells protects their telomeres from shortening to a critical length (9). The ectopic endometrium of women with EM may have specific aberrations, such as high TA levels, elevated hTERT gene expression, and increased mean endometrial telomere length (TL), compared with the endometrium of healthy women (10, 11). These abnormal ectopic endometrium-specific features are generally associated with a significant increase in telomeres and telomerase-related genes in the ectopic endometrium (12). These features also enhance the capacity of the endometrium to grow ectopically.

EM is also known as the “non-fatal cancer” because of its tumor-like biology of local infiltration, distant metastasis, and ease of recurrence. Much recent research has focused on the role of telomeres in tumor development. For example, telomere shortening may act as a tumor suppressor by preventing cell proliferation. Telomere shortening may also lead to widespread genomic instability, promoting cancer development (13, 14). However, no systematic studies have been conducted to clarify the role of telomere-related genes (TRGs) in the development of EM. Herein, a risk model was constructed using TRGs to predict EM, and then assessed the potential role of this risk model for, among other things, immune response.

## 2. Materials and methods

### 2.1. Data collection

RNA-seq data for EM were downloaded from the National Center for Biotechnology Information Gene Expression Omnibus (GEO) (https://www.ncbi.nlm.nih.gov/geo/) (15, 16). Data were collected and analyzed using R 3.6.3 software. The GEOquery package was used to download the Matrix file from the GEO database (17).

### 2.2. Data pre-processing and differentially expressed gene analysis

The TRGs were obtained from TelNet (http://www.cancertelsys.org/telnet/) (18), a database of genes involved in telomere maintenance, including a systematic assessment of the telomere maintenance machinery (TMM) signature and its inclusion in TMM pathway models or genome-wide mutational analyses of the cancer genome.

The R package sva was used to remove batch effects from different datasets (19). Inter-sample correction effects were calculated using principal component analysis (PCA) clustering. The R package limma (20) was used to screening for differentially expressed genes (DEGs) and telomerase-related genes. The package ggplot2 was used to plot volcanoes of DEGs to visualize differential expression patterns. Genes with adjusted P (Padj) <0.05 and |log_2_FC| > 1 were considered statistically significant.

### 2.3. Machine learning-based variable screening

Two machine learning methods, random forest model (RFM) and support vector machine (SVM)-recursive feature elimination (RFE), were used to screen for TRGs in EM. RFM is a classifier consisting of multiple decision trees, which ranks the importance of genes in regulatory relations. The SVM-RFE algorithm identifies the optimal variables by eliminating the feature vectors generated by the SVM (21). Receiver operating characteristic (ROC) curves and area under the curve (AUC) were used to evaluate the models' screening powers, resulting in a final set of EM telomere-associated signature genes.

### 2.4. Establishment and clustering of telomere-associated gene sets

A nomogram model was developed using the rms and rmda software packages to assess the predictive power of EM signature genes. Model accuracy was assessed using calibration curves, the Hosmer–Lemeshow test, clinical impact curve (CIC), and decision curve analysis (DCA) (22).

Unsupervised cluster analysis was applied to identify the different telomere gene subtypes and to classify samples for further analysis (23). The consensus clustering algorithm determined the number of clusters and their stability. The R package ConsensuClusterPlus was used to perform these steps, with 1,000 replications to ensure classification stability (24).

### 2.5. Establishment of C57BL/6 experimental mouse endometriosis model

C57BL/6 mice (specific pathogen-free, female) were obtained from the Department of Laboratory Animals, Capital Medical University. The animal experiment protocol used herein was approved by the Animal Ethical Use Committee of Capital Medical University (Ethical Number: AEEI-2021-219). Five C57BL/6 mice were housed in a well-controlled, pathogen-free environment in a barrier unit with a regulated light/dark cycle (12/12 h, 23–25°C). One mouse was used as the endometrial tissue donor after euthanization. The donor uterus was evenly divided into 8 endometrial fragments of 2 mm diameter using a disposable biopsy punch (2 mm) (Integra Miltex, Shanghai, China). These were injected into the peritoneal cavities of anesthetized mice (~100 mg tissue/0.5 mL PBS per mouse) *via* a 2 ml syringe. All mice survived the duration of the experiment. There were no significant between-mouse growth rate differences. Mice were euthanized 4 weeks later and both EM and uterine tissues were collected for immunohistochemical analysis.

### 2.6. Immunohistochemistry

Mouse EM and uterine specimens were fixed in 4% neutral paraformaldehyde solution, followed by paraffin embedding. Serial sections (5 μm) were then dewaxed. The fixed endometrial and EM lesion sections were incubated overnight at 4°C with anti-microtubule-associated protein (MAP) 7 (1:400, Servicebio, Wuhan, China), anti-grainyhead-like 2 (GRHL2) (1:100, Affinity, Changzhou, China), anti-retinoic acid receptor response protein 2 (RARRES2) (1:50, ABclonal, Wuhan, China), anti-ubiquitin carboxyl-terminal hydrolase-L1 (UCHL1) (1:1,000, Servicebio), anti-estrogen receptor alpha (ESR1) (1:1,000, Servicebio), and anti-protein reversionless 3-like (REV3L) (1:100, Affinity). Horseradish peroxidase-labeled goat anti-mouse IgG (immunoglobulin) (1:200, Servicebio) was then added and incubated for 50 min at room temperature. Sections were washed with distilled water, counterstained with hematoxylin, dehydrated, and mounted for microscopy. Colored areas were quantified using SlideViewer and Image Pro Plus software. The whole tissue sections were scored for staining intensity and percentage. The scoring scale was: 0 (no staining), 1 (light brown staining), 2 (brown staining), and 3 (dark brown staining). The percentage of positive cells was graded into one of 4 levels: 1 (<5%), 2 (5–30%), 3 (31–60%), 4 (61–100%). Immunohistochemistry staining score was calculated as follows: intensity score × percentage score.

### 2.7. Gene set enrichment analysis

An ordered list of genes was generated based on correlations between all genes and telomerase-related gene expressions using Gene Ontology (GO), Kyoto Encyclopedia of Genes and Genomes (KEGG), and Gene Set Enrichment Analysis (GSEA). The GO knowledgebase is the world's largest gene function information source (http://geneontology.org/) (25–27). KEGG is a collection of databases dealing with genomes, biological pathways, diseases, drugs, and chemical substances (www.kegg.jp/kegg/kegg1.html) (28–30). DEGs were defined by an absolute fold change >1.5 and Padj <0.05.

The GSEA computational method allows determination of overrepresented classes in large sets of genes or proteins that may be significantly association with disease phenotypes (31). The predefined gene set is from the MSigDB database (https://www.gsea-msigdb.org/gsea/msigdb/index.jsp) (32). Herein, an ordered list of genes was generated based on the correlation between all genes and telomerase-related gene expressions using GSEA. Enriched pathways were determined based on *P*-values and normalized enrichment scores.

### 2.8. Gene expression–immunity correlation

The Alizadeh Lab CIBERSORT analytical tool was developed by Newman et al. to estimate the abundances of member cell types in a mixed cell population using gene expression data (33). It was used herein to assess the relative proportions of the 24 immune infiltrating cells in the telomerase and gene clusters of EM samples (33, 34). Correlation analyses were then performed between these 24 immune cells and critical genes.

### 2.9. Potential drug identifications

To identify potential EM therapeutic agents, DEGs were uploaded to the connective map (CMAP) database (https://clue.io/) (35). Enrichment analysis was used to screen for relevant drugs with therapeutic potential (36). A negative enrichment value generally indicates that the drug is more likely to treat the disease, and a larger absolute value means that it is more disease-specific. Based on this, we screened for drugs with enrichments of <−0.5.

## 3. Results

### 3.1. Data collection and de-batch processing

**Figure 1:**
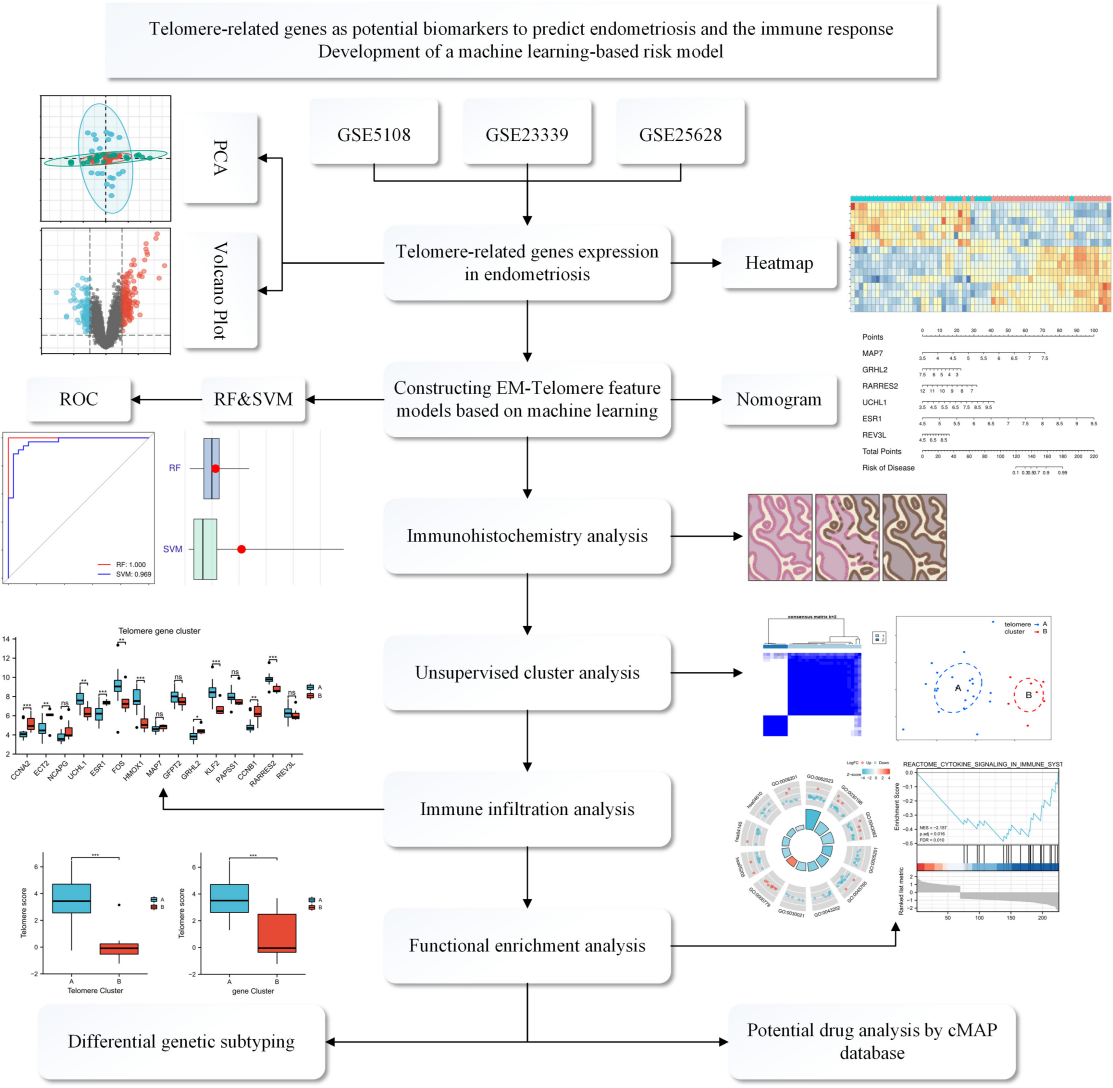
Flowchart of the TRGs as potential biomarkers to predict EM and immune response. We first selected three GEO databased EM datasets: GSE5108, GSE23339, and GSE25628. These data were normalized and then analyzed, deriving differential genes associated with telomeres. Then, a telomere gene signature model for EM was developed based on a machine learning approach and the model's predictive performance was evaluated. We also established a mouse model of EM and validated it at the immunohistochemical level for the derived differential genes. Meanwhile, functional enrichment analysis and immune infiltration analysis were performed to explain the pathways enriched by the differential genes and the associated immune responses. Finally, possible potential therapeutic agents were interpreted through the CMAP database.

The study flowchart is shown in Figure 1. Using key words “endometriosis, Homo sapiens,” the GEO database expression profile was searched and the following datasets were selected for inclusion:

A: GSE5108: 11 normal and 11 disease samples; sequencing platform: GPL2895 (37).
B: GSE23339: 9 normal and 10 disease samples; sequencing platform: GPL6102 (38).
C: GSE25628: 14 normal and 7 disease samples; sequencing platform: GPL571 (39).

**Figure 2:**
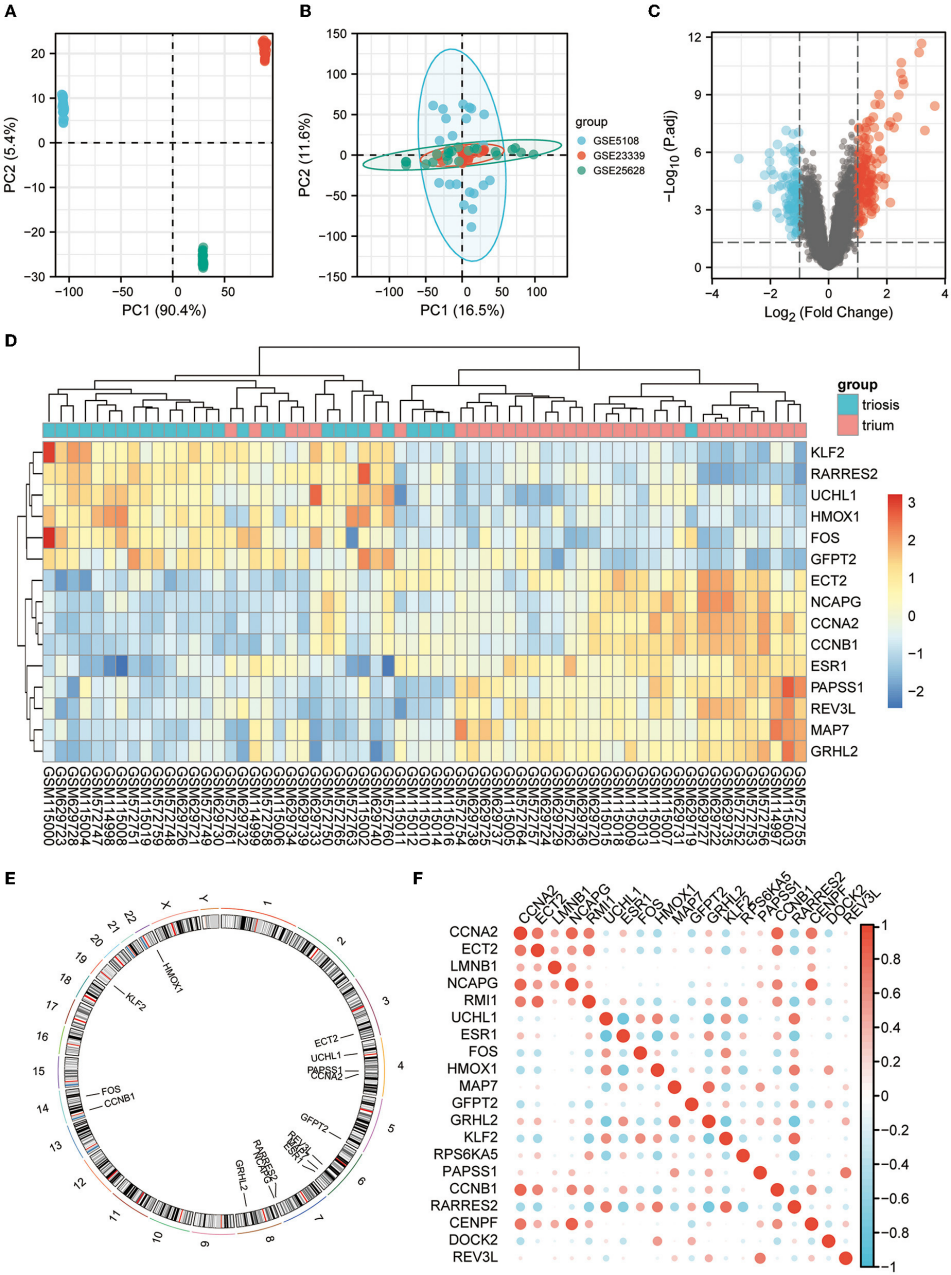
EM GEO dataset acquisition and telomere-related differential gene expression. **(A)** PCA plots for datasets GSE5108, GSE23339, and GSE25628 before sva correction. **(B)** PCA plots of the sva-corrected dataset. **(C)** Volcano map of EM-telomere DEGs. **(D)** Heatmap of 15 telomere-associated DEGs in EM and normal samples. **(E)** Circles of 15 telomere-associated DEGs expressed on chromosomes. **(F)** Correlation heat map of the expression profiles of 15 telomere-associated DEGs.

All three are mRNA-seq data GEO datasets, with large sample sizes and containing human EM and normal endometrial control samples. The SVA algorithm was used to batch-correct the three datasets and combine them into a single dataset of 35 normal and 28 EM samples. PCA was performed before and after removing the batch effect removal (Figures 2A, B).

### 3.2. Differential expression gene analysis

We screened for DEGs between the disease and control groups, and created a volcano plot of the results (Figure 2C). A total of 2,093 TRGs were obtained from TelNet, including 165 validated genes, 923 predicted genes, and 1,005 screened genes. Further screening of genes differing between the disease and control groups was performed based on TRGs. The top 15 genes with the most significant differences are shown in the heatmap (Figure 2D). The positions of these DEGs were then labeled on the chromosomes. The top three genes with the most significant differences, ESR1, RARRES2, and MAP7, were on chromosomes 6 and 7 (Figure 2E). Further analyses revealed correlations among these DEGs (Figure 2F).

### 3.3. Telomere-related signature models developed *via* machine learning

**Figure 3:**
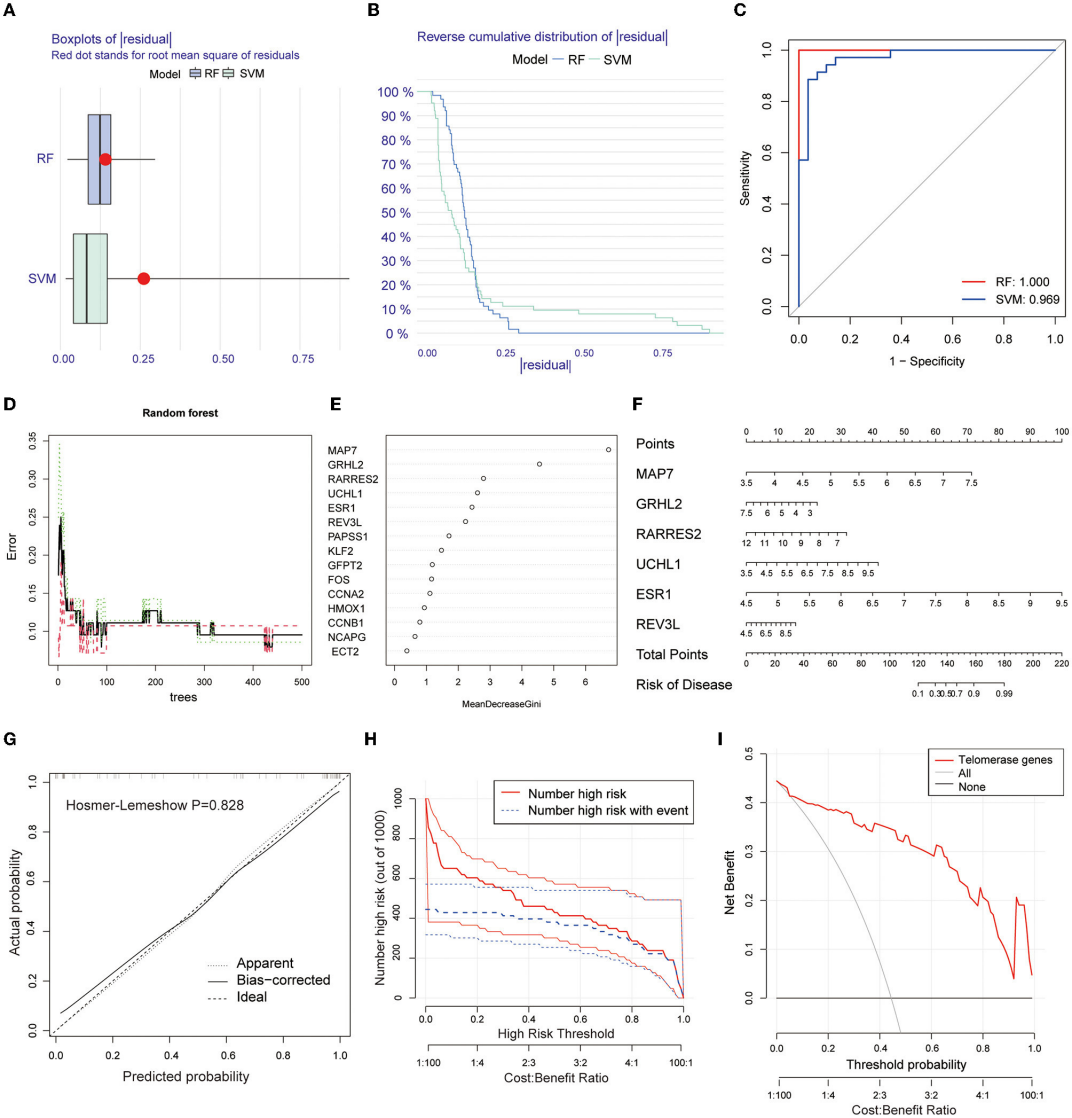
Screening telomere-related signature genes using machine learning methods, building nomogram prediction models, and evaluating model predictive power. **(A)** Box plot of residuals for RF and SVM models. Red dots are root mean squares of residuals. **(B)** Reverse cumulative distribution of residuals. **(C)** ROC plots for RF and SVM prediction models (AUC-RF = 1.0, AUC-SVM = 0.969). **(D, E)** RFM screening of 15 candidate genes. **(F)** Nomogram prediction model based on screening six EM-telangiectasia signature genes: MAP7, GRHL2, RARRES2, UCHL1, ESR1, and REV3L. **(G)** Calibration curve. X-axis is predicted disease risk. Y-axis is actual disease risk. Dashed diagonal line is perfect prediction of ideal model. Solid line is nomogram prediction model performance and fit to the dashed diagonal line is the model's predictive power (Hosmer–Lemeshow *P* = 0.828). **(H)** CIC. Red curve is number of people classified as positive (high risk) by prediction model at each probability threshold. Blue curve is number of true positives at each probability threshold. **(I)** DCA. CIC and DCA show good model predictive power when the risk threshold is from 0 to 0.92.

Two machine learning algorithms, RF and SVM, were used to screen telomere signature EM genes, plot box plots, and reverse cumulative distributions of residuals (Figures 3A, B). ROC curves show that RF (AUC = 1.0) had better predictive power compared with SVM (AUC = 0.969) (Figure 3C).

Based on the RFM, an importance score >2.0 was used as the threshold value. A total of 6 genes were included in the characteristic gene model: MAP7, GRHL2, RARRES2, UCHL1, ESR1, and REV3L (Figures 3D, E). A nomogram model was constructed based on the six included signature genes to predict disease risk (Figure 3F). Next, we validated the accuracy and predictive power of the model using CIC and DCA, with the Hosmer–Lemeshow test (*P* = 0.828) (Figure 3G). The CIC (Figure 3H) and DCA (Figure 3I) show good model predictive power at risk thresholds from 0 to 0.92.

### 3.4. Endometriosis mouse model and immunohistochemical analysis

**Figure 4:**
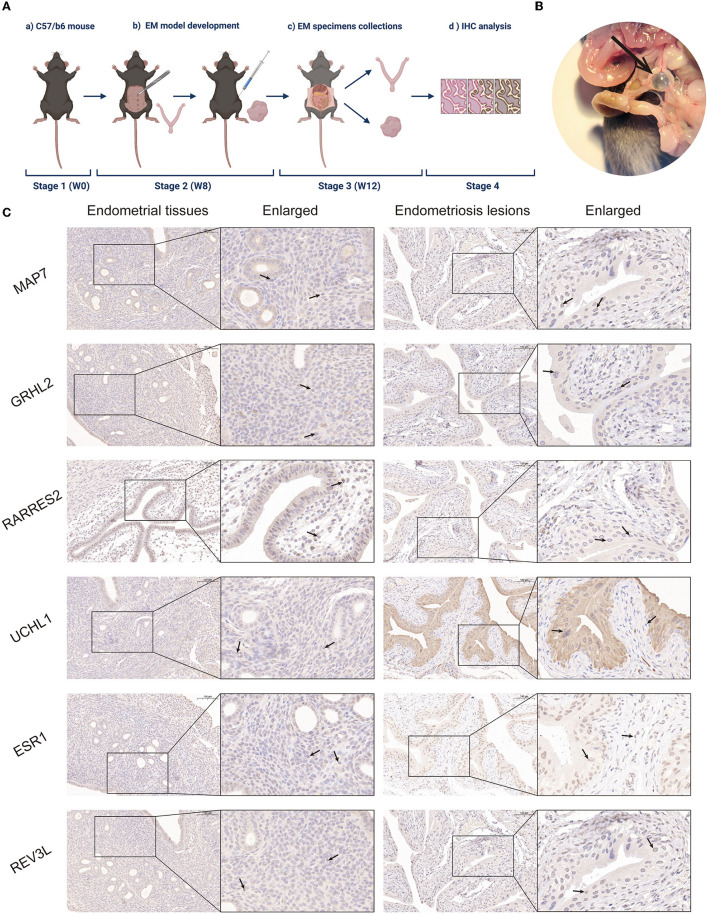
Establishment of a mouse EM model and immunohistochemical analysis. **(A)** Mouse model flowchart (BioRender^©^). **(B)** Fourteen days post-intraperitoneal implantation, endometrium-like lesions in the mesentery were visible under light microscopy in mice injected with endometrial fragments. Adhesions and vascular formations around the endometrial implants were observed. **(C)** Representative MAP7, GRHL2, RARRES2, UCHL1, ESR1, and REV3L staining of endometrial tissues and EM lesions in the mouse model.

To validate the role of the six screened genes in EM, an EM mouse model was developed (see flowchart in Figure 4A). Fourteen days after intraperitoneal implantation, mice injected with endometrial fragments developed endometrioid lesions in the intestine, mesentery, and peritoneum. Adhesions and vascular formations around the endometriotic implants were also detected (Figure 4B). Endometrial and endometriotic lesion tissues were collected for immunohistochemical analysis. Immunohistochemistry results further confirmed our database-based analysis (Figure 4C).

### 3.5. Identification of telomere gene subtypes and immune infiltration in endometriosis

**Figure 5:**
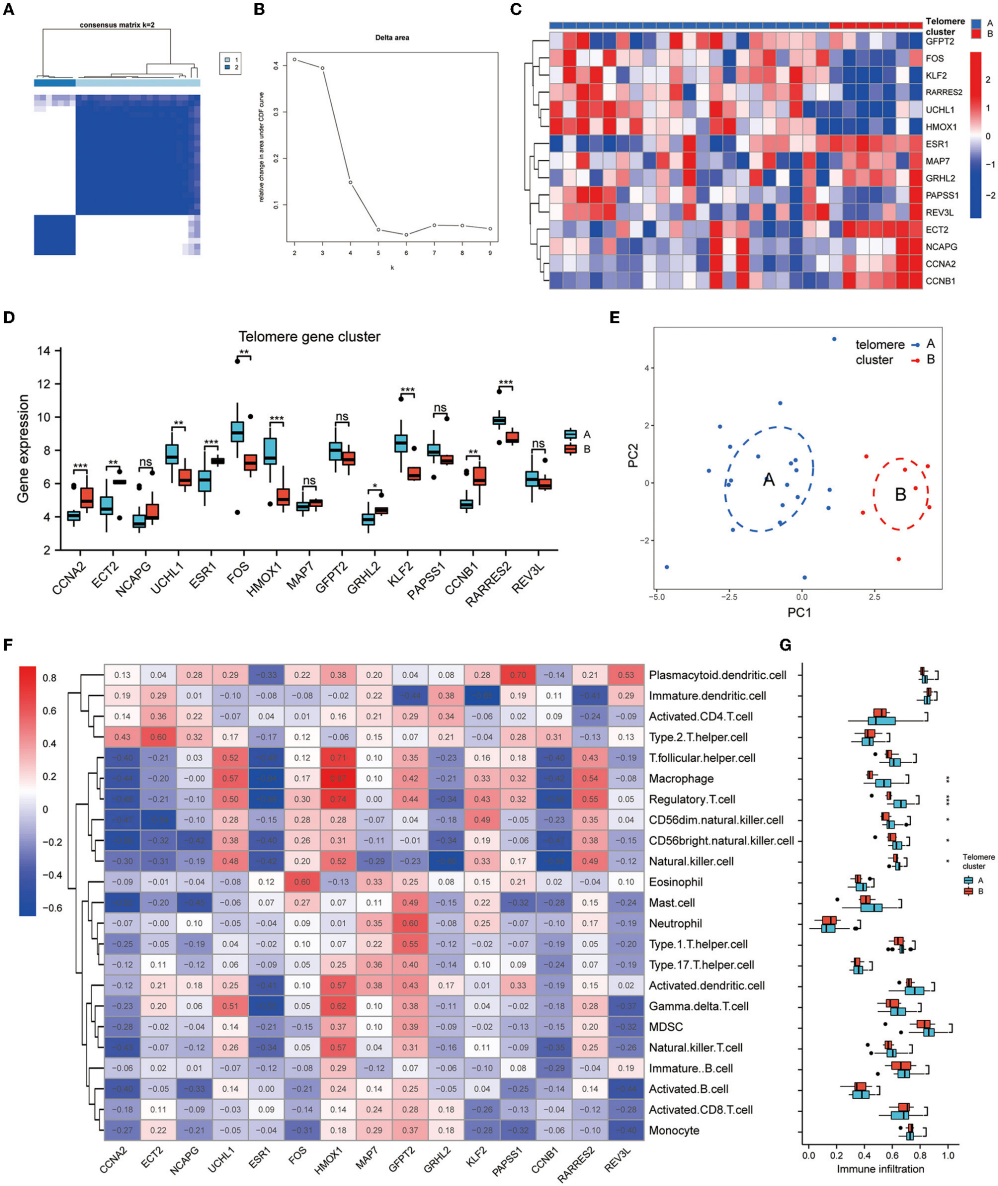
Identification of telomere gene subtypes and immune infiltration in EM. **(A, B)** Two telomere gene modification subtypes identified in EM by setting K values in range 2–9 and selecting the optimal K = 2. **(C)** Heatmap of telomere differential genes in EM differentially expressed in clusters A and B. **(D)** Boxplot of differential telomere gene expressions in clusters A and B in EM. **(E)** PCA analysis shows that clusters A and B can distinguish samples well. **(F)** Heatmap of EM telomere-related differential genes in relation to immune cells according to ssGSEA analysis. **(G)** Boxplot of differential expressions of immune cells in clusters A and B based on ssGSEA analysis. ^*^P < 0.05, ^**^P < 0.01, ^***^P < 0.001.

To investigate the modification patterns of telomerase genes in EM, we performed an unsupervised consensus clustering analysis of 28 EM samples based on the expressions of 15 telomere gene regulators. Two telomere gene modification subtypes in EM were identified by setting K value ranges at 2–9 and selecting the optimal K = 2 (Figures 5A, B). Among them, clusters 1 and 2 contained 21 and 7 samples, respectively. PCA showed that these two subtypes could clearly distinguish the samples (Figure 5E). In addition, we identified 10 telomerase gene regulators, which were significantly differentially expressed in the two isoforms (Figures 5C, D).

The heat map of the immune cell correlation analysis is in Figure 5F. HMOX1 was positively correlated with macrophages, with the most significant correlation coefficient of 0.87. KLF2 was negatively correlated with immature dendritic cells, with the smallest negative correlation coefficient of −0.65. Further analysis of immune cell differences indicated that five different immune cells differed significantly between clusters A and B: CD56 bright natural killer cell, CD56dim natural killer cell, macrophage, natural killer cell, and regulatory T cell (Figure 5G).

### 3.6. Functional enrichment analysis of telomere signature gene sets

**Figure 6:**
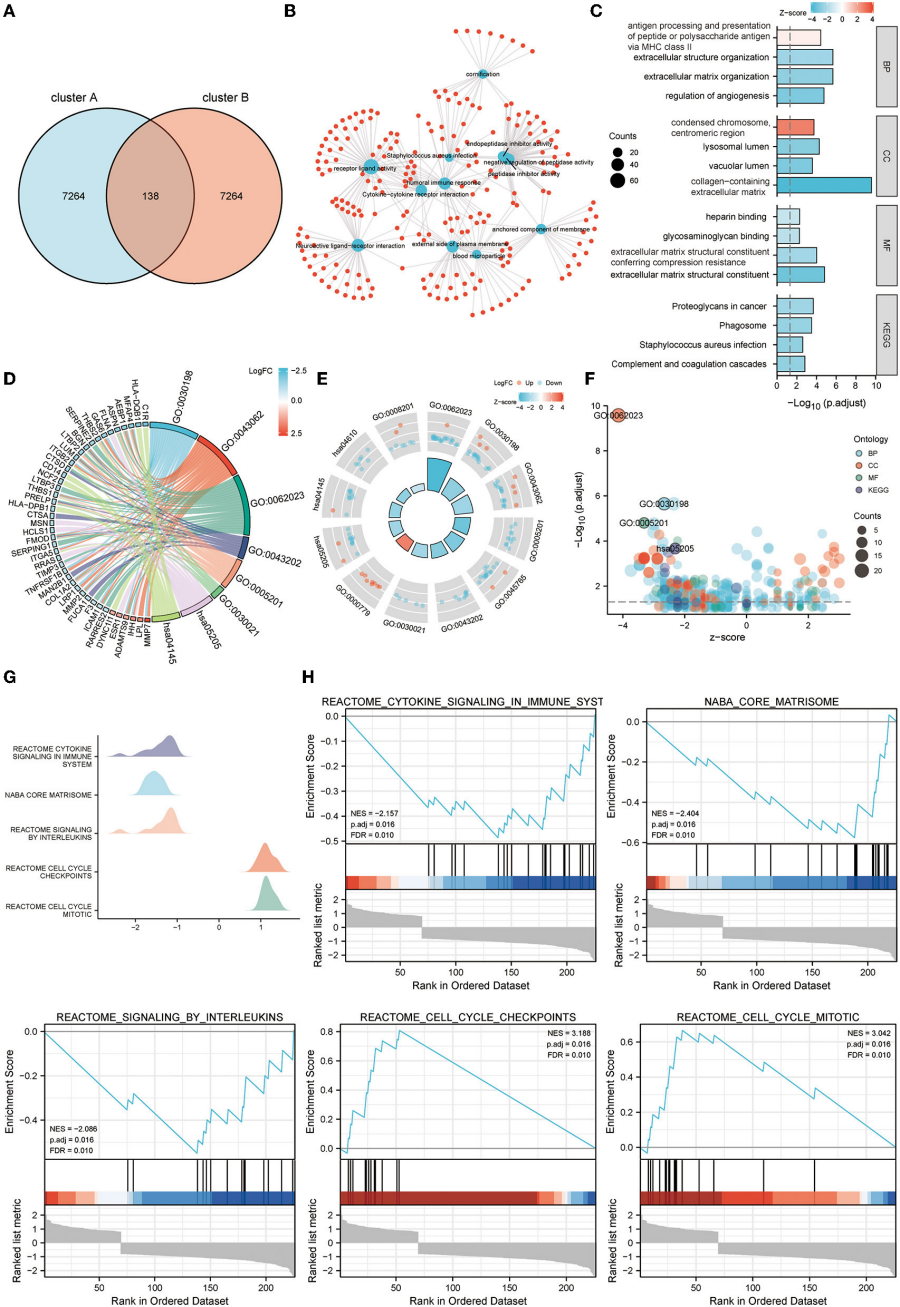
Functional enrichment analysis of telomere signature gene sets. **(A)** Venn diagram shows a total of 138 differential genes between clusters A and B. **(B–F)** GO and KEGG enrichment analysis of differential genes between clusters A and B, **(B)** network diagram, **(C)** column chart, **(D)** string diagram, **(E)** circle diagram, and **(F)** bubble diagram. **(G, H)** GSEA enrichment analysis of differential genes between clusters A and B, **(G)** mountain diagram, **(H)** GSEA enrichment results for the five most significant differential genes.

GO, KEGG pathway analysis, and GSEA were used to determine telomere gene roles and their potential mechanisms in EM. DEGs analysis was performed for both cluster A and cluster B samples (logFC >1, Padj <0.05) (Figure 6A). For the 138 DEGs obtained, we performed GO and KEGG analyses. Critical genes were mainly associated with extracellular matrix organization, extracellular matrix structural constituent, proteoglycans in cancer, and other pathways (Figures 6B–F).

Further GSEA analysis showed that critical genes were enriched in Reactome cytokine signaling in immune system, Naba core matrisome, Reactome signaling by interleukins, Reactome cell cycle checkpoints, Reactome cell cycle mitotic, and other pathways (Figures 6G, H).

### 3.7. Genetic subtypes of DEGs and immune infiltration analysis

**Figure 7:**
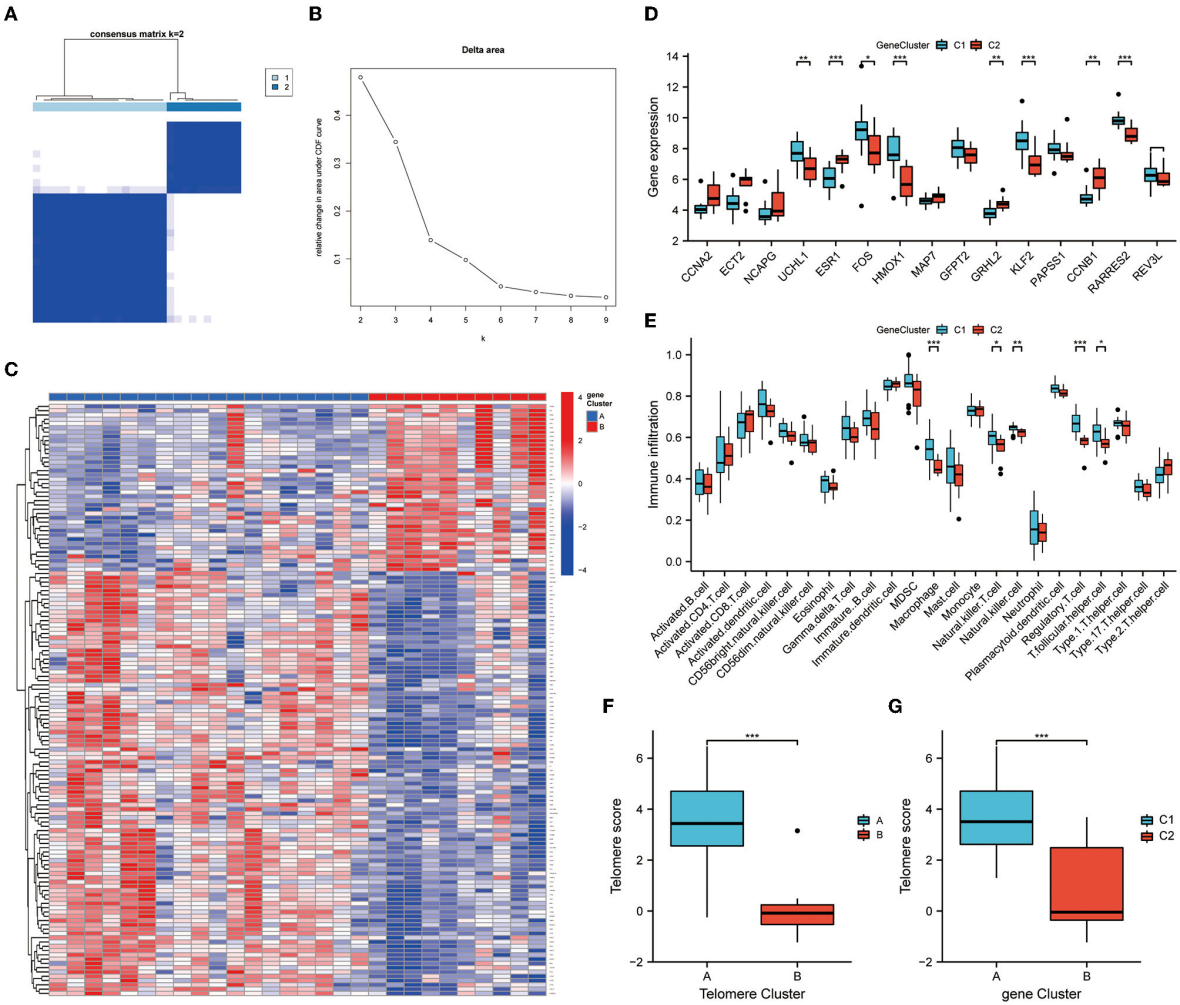
Genetic DEG subtypes and immune infiltration analysis. **(A, B)** Distinct isoforms of differential gene modifications identified by setting K values in range 2–9 and selecting the best K = 2. **(C)** Expression heat map of the 138 differential genes in clusters 1 and 2. **(D)** Expression of telomere genes in clusters 1 and 2. **(E)** ssGSEA demonstrates infiltration of immune cells in clusters 1 and 2. **(F, G)** Telomere gene scores were significantly upregulated in subtype A/subtype 1. ^*^P < 0.05, ^**^P < 0.01, ^***^P < 0.001.

We further subtyped the samples according to DEG expressions. An unsupervised consensus clustering analysis was performed on 28 EM samples, based on cluster 1 and 2 DEG expressions. Two distinct isoforms of differential gene modifications were identified by setting K values in the range from 2–9 and selecting the best K = 2 (Figures 7A, B). Clusters 1 and 2 contained 18 and 10 samples, respectively. Figure 7C shows the expression heat map of the 138 DEGs in clusters 1 and 2. Of the 15 telomere signature genes, eight were significantly differentially expressed in clusters 1 and 2: ESR1 (*P* < 0.001), HMOX1 (*P* < 0.001), KLF2 (*P* < 0.001), RARRES2 (*P* < 0.001), UCHL1 (*P* < 0.01), GRHL2 (*P* < 0.01), CCNB1 (*P* < 0.01), and FOS (*P* < 0.05) (Figure 7D). Further immune infiltration analysis revealed significant differences in the expressions of five immune cells in DEG analysis: macrophage (*P* < 0.001), natural killer T cell (*P* < 0.05), natural killer cell (*P* < 0.01), regulatory T cell (*P* < 0.001), and T folic helper cell (*P* < 0.05) (Figure 7E).

Finally, PCA was performed on the telomere gene scores for each sample. Telomere gene scores differed significantly between telomere genetic subtypes and differential genetic subtypes (*P* < 0.001). In both subtypes, telomere gene scores were significantly upregulated in subtype A/subtype 1. These results further demonstrate the accuracy and reliability of our model (Figures 7F, G).

### 3.8. Identification of relevant small-molecule compounds for endometriosis treatment

By screening the DEGs identified in the CMAP database, we identified potential therapeutic agents for EM treatment. Eleven potential small-molecule compounds were identified based on the screening criteria of normalized connective score <2 and log_10_q >15. Drug mechanism analysis suggested that crizotinib, AZD-4547, C-646, docetaxel, rociletinib, ENMD-2076, AMG-232, dioscin, mibefradil, NNC-05-2090, and nutlin-3 have potential as novel EM treatments (Supplementary Table 1).

## 4. Discussion

Recent clinical and basic research advances have updated our understanding of EM. While classic EM treatment options have included radical surgery and conservative pharmacology, improved molecular biology is giving us a better understanding of EM heterogeneity and directing a more precise search for novel prognostic markers and treatments.

Processing complex research data and building predictive models by machine learning methods can better predict clinical disease changes and has advantages in oncology and related non-oncology research (23). Machine learning algorithms are mainly divided into two categories: supervised learning algorithms for building predictive models and unsupervised learning algorithms for building descriptive models. The former include K-nearest neighbor method, neural network, SVM, and RFM (40); the latter include association rules and k-means clustering algorithms. Huang et al. proposed a novel data analysis method based on data augmentation and elastic data shared lasso regularization, which can infer and integrate information from multiple gene expression datasets (41). SLNL (self-paced learning network-based logistic regression model), a new method for gene selection and phenotype classification, is an absolute network-based logistic regression model that may be useful for tumor diagnosis and treatment (42). The cumulative evidence supports the non-trivial superiority of machine learning methods for improving disease diagnostic accuracy.

Herein, we screened and identified DEGs by analyzing three DEO database EM datasets and TelNet telomere-related gene sets. These DEGs contained six upregulated genes and nine downregulated genes. To identify signature genes that could be potential markers, we compared the screening ability of two different machine learning methods, RFM and SVM-RFE. Residual plots and ROC curves indicate that the RFM-built model had better predictive power. Subsequent analyses were based on the RFM method, to screen for six EM telomere signature genes: MAP7, GRHL2, RARRES2, UCHL1, ESR1, and REV3L. Our mouse model and further immunohistochemical analyses further validated the findings.

MAP7 promotes cervical cancer cell line migration and invasion, and epithelial-mesenchymal transition (EMT), by regulating autophagy (43, 44). GRHL2 is involved in EMT through CLDN4 core promoter and E-cadherin gene regulation (45). Gynecological tumor studies have also demonstrated that increased GRHL2 expression is associated with poor prognosis in ovarian cancer (46). RARRES2 is a small, secreted protein associated with a variety of cancers, and higher serum RARRES2 levels have been shown to be associated with improved overall survival in adrenocortical tumors (*P* = 0.0227) (47). The deubiquitinase UCHL1 is an oncoprotein that promotes the growth and progression of cancer cells. In specific types of breast cancer, high UCHL1 activity may be targeted to enhance the efficacy of endocrine therapy in estrogen receptor-negative breast cancer cells and slow migration and metastasis of triple-negative breast cancer (48, 49). UCHL1 may also serve as a biomarker for EM and a potential new therapeutic target.

A meta-analysis of 24 case control studies showed that ESR1 (TA)n gene polymorphisms were associated with susceptibility to EM (50). ESR1 rs9340799 was associated with EM-associated infertility and *in vitro* fertilization failure (51). REV3L, the catalytic subunit of DNA polymerase ζ, plays a vital role in the DNA damage tolerance mechanism of translocation synthesis. Cervical cancer studies have shown that inhibition of REV3L expression and overexpression, respectively, enhances sensitivity and resistance of cervical cancer cells to cisplatin (52). We developed a predictive nomogram model for EM based on the above six genes.

Although the etiology of EM is unknown, immune dysfunction has been suggested as a pivotal contributor to the growth of ectopic lesions of endometrial debris. Unsupervised cluster analysis based on our screened telomere genes showed that 10 differed significantly by cluster. Further immune infiltration analysis showed that macrophages, regulatory T cells (CD56^dim/bright^), natural killer cells, and other were significantly overexpressed in Cluster A (*P* < 0.05). This is similar to the ssGSEA results obtained after further genotypic clustering analysis. Lindsey et al. showed that the number of macrophages was significantly elevated in peritoneal fluid and ectopic endometrium in patients with EM (53). NK cells were present in the peripheral circulation and uterus, mainly as CD56^dim^CD16^+^ and CD56^bright^CD16^+^. This is consistent with our findings. Therefore, it has been hypothesized that NK cell dysfunction in EM contributes to the immune-based spread of ectopic endometrial debris to the peritoneal cavity (54).

Nevertheless, it is unclear whether this immune dysfunction is a cause or a consequence of EM development. Therefore, further studies are needed to determine whether immune dysfunction may be an EM treatment, and to further guide potential immunotherapy or targeted therapy.

Women with EM high TA levels, high hTERT gene expression and hTERT protein levels, and longer mean endometrial TL compared with the endometrium of healthy women (12). Enrichment analysis of telomere signature genes in EM showed that these DEGs are primarily involved in extracellular tissue, extracellular matrix, and angiogenesis pathways, and are associated with cell cycle and cytokine pathways. These results suggest that TRGs may mediate the development of EM by mediating biological pathways such as EMT and angiogenesis.

Screening for target drugs is another vital aspect of EM research. Eleven drugs were screened herein. Of these, crizotinib has been approved for advanced anaplastic lymphoma kinase-positive lung cancer (55, 56). Docetaxel is indicated for the treatment of locally advanced or metastatic breast and non-small cell lung cancers (57). Clinical trials are also ongoing for some of the other screened drugs, which may act in mechanistic pathway of TRGs in EM.

## 5. Conclusion

This study shows, for the first time, relations between TRGs and EM development, and establishes a nomogram model of characteristic genes. The immune infiltration profile and associated sensitive drugs were also analyzed, which may provide practical value for future studies targeting telomeric genes in EM treatment. These findings shed new light on the role of TRGs in EM. Telomere-associated genes also enhance our understanding of the mechanisms involved in the susceptibility of EM to recurrence and malignant changes. Our ongoing work will focus further on the specific targets of TRGs in EM and their immune infiltration-related mechanisms. We will conduct more in-depth *in vivo* and *in vitro* validation experiments.

## Data availability statement

The datasets presented in this study can be found in online repositories. The names of the repository/repositories and accession number(s) can be found in the article/Supplementary material.

## Ethics statement

The animal study was reviewed and approved by Animal Ethical Use Committee of Capital Medical University.

## Author contributions

HZ and WK contributed significantly to data analyses and wrote the manuscript. WK contributed to the conception of the study. SC, ZP, XZ, YX, and DL helped perform the analyses and contributed to constructive discussions. All authors read and approved the final manuscript.

## Supplementary material

Supplementary Table 1 Screening of 11 potential EM therapeutic agents according to the CMAP database, mechanism of action, and normalized connective score.
